# Progress in Medicine

**Published:** 1898-02-12

**Authors:** 


### PROGRESS IN MEDICINE.

### NEUROLOGY.

**Sunstroke.**—In the month of August, 1896, over 2,000 cases of death from sunstroke were recorded in the Central and Atlantic States of America, and during the following year a number of valuable papers on the subject were published.

Phillips finds from a careful investigation of the meteorological conditions, that the daily mean temperatures during the four days August 9th-12th—those of the greatest number of sunstrokes—was from 10 deg. to 13 deg. above the normal for the month of August, and the absolute humidity was also at its maximum, whilst the relative humidity was above the normal in the Central, below the normal in the Middle Atlantic States. These conclusions only differ from those generally accepted in regard to the influence of the relative humidity. The degree of atmospheric temperature necessary or likely to give rise to sunstroke varied. For instance, sunstrokes were reported in Boston when the average temperature of the day reached 82 deg., or 13 deg. above the August normal, whilst the normal August temperature of New Orleans was 82 deg., and no sunstrokes occurred. Evidently sunstrokes are not excited by the same degree of temperature in every locality. On further comparison, however, it appeared that sunstroke becomes imminent during the summer months when the mean temperature of any one day, or of several consecutive days, becomes equal, or nearly equal, to the normal maximum temperature for the period.

Lambert,[2] from an analysis of 805 cases of sunstroke admitted into New York hospitals in August, 1896, says that clinically the cases can be divided into the following groups: (1) Heat prostration: Amongst the prodromes of which were severe headache, dizziness, pain in back and legs or epigastrium, sometimes numbness and tingling of hands and feet—these might come on from one to three days before admission, or might appear suddenly. The patients did not lose consciousness, and the temperature did not go above 105 deg. Fahr.; in a few it was subnormal. (2) The asphyxial or milder form of sunstroke. Prodromes varied, or might be absent, and the patient somewhat suddenly lost consciousness, with a temperature of 105 deg. or lower. The unconsciousness lasted until after a bath, or perhaps 24 hours. (3) The hyperpyrexial form—by far the most serious, with a mortality of 25 per cent. All the patients had a temperature of 105 deg. or over, and the majority lost consciousness. In every case of a temperature of 110 deg. Fahr., or over, coma was complete. During the bath or cold pack, or within an hour afterwards, about one-half of the patients became conscious; but many did not regain consciousness until next day, or until from three to five days. Many with a temperature of over 108 deg. did not regain consciousness at all; the temperature dropped, but the pulse remained frequent, dyspnœa and cyanosis were often marked, and they died. The fall in temperature in the majority of cases was followed by one or more secondary rises, which often required active treatment. The fatal complications observed were meningitis, pneumonia, and acute exacerbation of chronic kidney disease. The cause of death in most cases was failure of the respiratory and cardiac centres. Treatment in the cases of heat-prostration and asphyxial cases was by baths of 60 deg. Fahr., or sponge baths; and all recovered. The hyperpyrexial cases were all treated by the application of cold, but the exact method and temperature of the water applied varied. By far the best results—a mortality of 12 per cent., as compared with the general one of 25 per cent. in this class of cases—were obtained at St. Vincent's Hospital, where the method employed was to cover the patient with a cotton sheet, and repeatedly dash him with cold water until the temperature fell to 104 deg. or 103 deg. The patient was then wrapped in blankets and surrounded by hot bottles. This warm pack brought on sweating, and the body temperature fell slowly to normal. If a second rise of temperature occurred the procedure was repeated. Kinnear[3] is of opinion that the best treatment for heat prostration is the application of cold to the spine by means of a bag 4 in. wide and 22 in. long, packed with fragments of ice. He also says that by this means he has been enabled to obtain refreshing sleep during the hottest summer nights, and advocates its use as a prophylactic measure against heat exhaustion and sunstroke during hot weather.

**Optic Neuritis in Cerebral Tumour.**—Martin[4] has collected the records of 600 cases of cerebral tumour and analysed them to ascertain whether the occurrence of optic neuritis has any localising value or not. He finds that the male sex is more than twice as liable as the female to intracranial growths. The position of the headache is of no value in determining that of the tumour; in many cases of tumour of the motor area and corpus callosum headache was absent, occipital headache was frequent in cerebellar tumour, whilst frontal headache may occur in all kinds of tumours. Optic neuritis is constantly (100 per cent.) present in tumour of the corpora quadrigemina, so that in the absence of optic neuritis we may exclude this position for the tumour. The cerebellum and parieto-occipital region come second, with optic neuritis in 89 per cent. of cases. On the other hand, optic neuritis is found in a comparatively small proportion of cases of tumour of the corpus callosum, pons, and medulla. When optic neuritis is more marked in one eye than in the other, the seat of the lesion is probably on the side on which the neuritis is more marked in the proportion of two to one. With regard to the nature of the tumour, it was found that optic neuritis is most often absent if the tumour is tuberculous, and is most frequently present if the tumour is a glioma, cystic, or a hydatid. The most common form of intracranial growth is sarcoma, whilst gliomata and tuberculous tumours are of about equal frequency, but less common than sarcomata.

**Chorea.**—Riesman[5] points out the various types of chorea in adult and advanced life which have been described. They have been divided by Hersingham into the following groups: (1) One resembling chorea minor of childhood, and lasting from a week or two to several months; (2) one due to a coarse subcortical lesion which follows hemiplegia, and sometimes precedes it; (3) the hereditary form of chorea, or Huntington's chorea; and (4) an incurable form, without hereditary basis, and not due to subcortical lesion.

Chorea minor in the aged was first described by Graves. There is not the same tendency to cardiac complications as in juvenile chorea, nor is rheumatism so frequent in the personal history of the patient. It may occur as late as the age of 86 years. Graves remarked in relation to these cases, "Thus do diseases of the nervous system affect a second childhood."

Post-hemiplegic chorea, first described by Weir Mitchell, and then by Charcot, is characterised by choreiform movements of moderate severity, intensified by voluntary effort, and affects especially the distal parts of the extremities, chiefly the fingers and toes. The movements are slow and somewhat athetoid in nature. Hemiplegia is nearly always present, and in some cases is associated with hemianæsthesia. The reflexes are exaggerated on the choreic side. The lesion giving rise to post-hemiplegic chorea is situated subcortically, usually in the neighbourhood of the optic thalamus, or within the posterior limb of the internal capsule. In rare cases the chorea precedes the occurrence of hemiplegia.

In Huntington's chorea, the essential features are the onset of choreiform movements in adult life, a progressive dementia, and the existence of the disease in the preceding generation. The movements are very incoordinate and more rhythmical than in chorea minor, and usually, particularly in the early stages, are under the control of the will, and are nearly always bilateral. Speech is almost invariably affected.

The fourth class of cases differs from chorea minor in its incurability, and from Huntington's chorea in the absence of hereditary bias, and in the absence of gross subcortical lesion. A question as yet unsettled is whether these cases belong to a separate type from Huntington's chorea. It would seem that the existence of a hereditary bias induces the development of the disease at an earlier period of adult life, and predisposes to dementia and other forms of mental failure; in the absence of heredity the onset is later in life, and mental disease is not so frequent, only occurring in two-fifths of the cases.

The following practical conclusions result from consideration of the above facts: If chorea comes on in adult life or in old age, and is not post-hemiplegic in character, it may be Huntington's chorea, or chorea minor, or chronic progressive chorea. In Huntington's chorea there will be a hereditary history, the prognosis is unfavourable, and the case will end in dementia. If there is no hereditary history the case is one of either chorea minor, or chronic progressive chorea; but it is impossible to say which it will turn out to be; it may turn out to be chorea minor, in which case it will terminate in recovery in a few weeks or months; if it lasts more than that time the case is one of chronic progressive chorea, which is incurable, though the probability of mental failure occurring is not nearly so great as in Huntington's chorea.

Michell Clarke[6] gives a good account of a case of Huntington's chorea, in which there was a marked family history. In a brother of the patient, who had chorea and dementia, and who died from pneumonia, examination of the brain showed no naked eye alteration, but on microscopical examination there was widespread, though partial, degeneration of the cells of the cerebral cortex, especially in the second and third layers, and an increase of the amount of interstitial tissue and of neuroglia cells. Clarke remarks that similar changes have been observed in cases of ordinary chorea, and the identical nature of the spasms in the two conditions would seem to indicate a similar affection of the same parts of the nervous system in both cases.

**[illegible]tment of Chorea Minor.**—Overend[7] comes to the following conclusions as to the value of arsenic and belladonna in the treatment of chorea minor. Belladonna appears to be most beneficial in recent cases, and its influence is sometimes very marked in severe forms. In obviously rheumatic cases arsenic in large doses may be given a trial, or may be combined with belladonna from the first. As much as 30 minims of belladonna tincture every four hours may be given if the patient is carefully watched; it should be discontinued if marked symptoms of belladonna poisoning occur, or if no improvement is effected within ten days; as a rule, its influence on the movements makes itself felt in about four days. As soon as the movements become trivial, or occur only on exertion, it is better to omit the belladonna and to commence massage of the affected muscles, and to give cod-liver oil and syrup of phosphate of iron or other tonics.

**Habit Chorea.**—Sinkler[8] says that there are two varieties of habit chorea. The first is one in which the disease is the result of a trick or habit in the child or adult, while in the second class the affection is due to some predisposing cause, such as is operative in the production of Sydenham's chorea. The movements in habit chorea are unlike the spasmodic twitching which occur in the different varieties of tic (tic convulsif), as may be easily seen if the movements affect the face, which is the region selected in the majority of cases. There may be blinking of the eyes, or a sudden contraction of the orbicularis palpebrarum, or a depression and elevation of the eyebrows. A contraction of the zygomatic muscles is a common symptom. Movements of the head often occur; in other cases there is shoulder-shrugging. Often habit chorea arises from nasal diseases, which give rise to sniffling or distortion of the facial muscles. In the greatest number of cases, however, some ocular defect, either a refraction error or disease of the lids, is responsible for unnatural blinking, orbicularis contractions or movements of the upper facial muscles. The prognosis depends largely on the length of time the affection has lasted before the patient is seen; if seen early enough the disease may be cured in a great proportion of cases. In adults in whom the disorder has lasted some time it is difficult, if not impossible, to arrest the movements, for habit chorea has not only a tendency to become uncontrollable but also to increase its range and involve other groups of muscles. The treatment consists in firstly correcting any ocular or nasal defect which may have set up the disorder, and secondly in administering drugs. In nearly all cases the general health is at fault, the patient is anæmic or run down from overstudy, improper food, or some attack of illness. The one drug which has a special influence on the disease is arsenic, which should be administered in increasing doses until some toxic influence is observed.

Dyce Duckworth[9] has recorded some fresh cases of cerebral disease in which the function of respiration entirely ceases before that of the circulation. It has been experimentally demonstrated that increased intracranial pressure slows the pulse and respiration, and finally causes arrest of the latter, the heart still continuing to beat. Analogous clinical phenomena have been observed. Thus a girl aged 15 years had suffered from otitis media for a year; cerebellar abscess followed. She was trephined, pus was sought, but not found. The breathing ceased, and artificial respiration was continued for four hours before the circulation stopped. Patients suffering from cerebral hæmorrhage, cerebral tumours, depressed fractures of the skull, and sudden and violent concussion, especially when applied in the occipital region, may die in this manner from respiratory failure. The clinical lessons are that efforts should promptly be made to relieve intracranial pressure by trephining, whilst artificial respiration should be diligently maintained until it can resume its function independently.

[1] Phillips, Internat. Med. Mad., Aug., 1897. [2] Lambert, Med. News, July, 24, 1897. [3] Kinnear, Med. Record, Aug. 21, 1897. [4] Martin, Lancet, July 10, 1897. [5] Riesman, Amer. Jour. Med. Sc., Aug., 1897. [6] Michell Clarke, Brain, 1897. [7] Overend, Lancet, July 31, 1897. [8] Sinkler, Amer. Jour. Med. Sc., May, 1897. [9] Dyce Duckworth, Lancet, Sept. 4, 1897.

